# Acute and repeated toxicological study of Myelophil, an ethanol extract of a mixture of *Astragali Radix* and *Salviae Miltiorrhizae Radix*, in beagle dogs

**DOI:** 10.1186/s12906-019-2588-3

**Published:** 2019-07-08

**Authors:** Jin-Yong Joung, Jin-Seok Lee, Jung-Hyo Cho, Dong-Soo Lee, Chang-Gue Son

**Affiliations:** 1 Liver and Immunology Research Center, Oriental Medical Collage of Daejeon University, 75, Daedeok-daero 176beon-gil, Seo-gu, Daejeon, 35235 Republic of Korea; 2 Department of Internal Medicine, Daejeon St. Mary’s Hospital of Catholic University, Daejeon, Republic of Korea

**Keywords:** Myelophil, Astragali Radix, Salviae Miltiorrhizae Radix, Safety, Toxicological study, NOAEL, HED

## Abstract

**Background:**

To evaluate the pharmaceutical safety of Myelophil, an ethanol extract of a mixture of *Astragali Radix* and *Salviae Miltiorrhizae Radix*, using both acute and repeated toxicological studies.

**Methods:**

A total of 40 beagle dogs (20 each male and female) were fed doses up to 5,000 mg/kg for the acute study and up to 1,250 mg/kg for the 13-week repeated dose toxicological study. Adverse effects were examined intensively by comparing the differences between normal and drug-administered groups using clinical signs, autopsies, histopathological findings, hematology, urinalysis, and biochemical analysis.

**Results:**

No mortality or drug-related clinical signs were observed in the Myelophil-treated groups, except for vomiting due to an excessive dose (5,000 mg/kg). Likewise, in the repeated toxicity test, compound-colored stools in the Myelophil-treated groups and soft stools in all groups, including the control, were observed. No drug-related abnormalities were found in the histopathology, hematology, urinalysis, and biochemical analyses for any doses of Myelophil.

**Conclusion:**

These results support the safety of Myelophil with a no observed adverse effect level (NOAEL) of 1250 mg/kg in beagle dogs, which corresponds to a human equivalent dose (HED) of 694 g/kg.

**Electronic supplementary material:**

The online version of this article (10.1186/s12906-019-2588-3) contains supplementary material, which is available to authorized users.

## Background

The use of complementary and alternative medicine is increasing worldwide, and many medicinal plants are being used for disease treatment and health improvement purposes [1, 2]. Since medicinal plants are derived from nature and have been used for a long time, they are generally regarded as safe [3]. However, recent studies have warned about the safety of medicinal plants [4, 5]. In particular, the potential hepatotoxicity and renal toxicity of medicinal plants have been reported [6, 7].

Myelophil is a 1:1 mixture of the 30% ethanol extracts of *Astragali radix* and *Salvia radix* and is used clinically to treat patients with chemotherapy/radiation therapy-induced myelosuppression or chronic fatigue-related disorders [8, 9]. In particular, in addition to the results in an animal model, Myelophil showed anti-fatigue effectiveness in a clinical trial on idiopathic chronic fatigue [9, 10]. In addition, a previous preclinical study showed partial evidence for the safety of Myelophil from a sub-chronic toxicological study using Sprague Dawley (SD) rats [11]. Regarding the wide spectrum of Myelophil applications with respect to age, period and subjects, there is a strong demand for further evidence on the safety of Myelophil. In particular, comparisons with non-rodent-derived studies are needed due to the limitations of rodent-based toxicity studies [12].

Individually, both *Astragali radix* and *Salvia radix,* which compose Myelophil, have been reported to be safe in several toxicological studies [13, 14]. These medicinal plants are known as two representative herbs used to treat *Qi*- and *Blood*- disorders, respectively, and they are frequently prescribed together in a formula in clinical practice [9]. However, to date, no non-rodent animal studies have been conducted to evaluate the safety of the combination.

Myelophil is an anti-fatigue therapeutics candidate, which its effects will be evaluated via clinical study in the future. In order to provide the safety evidence according to Korea Food and Drug Administration (KFDA), this study aimed to evaluate the tolerance range in a single acute study and to estimate the no observed adverse effect level (NOAEL) of Myelophil using a 13-week repeated toxicological test on beagle dogs.

## Materials and methods

### Preparation and fingerprinting of Myelophil

**Fig. 1:**
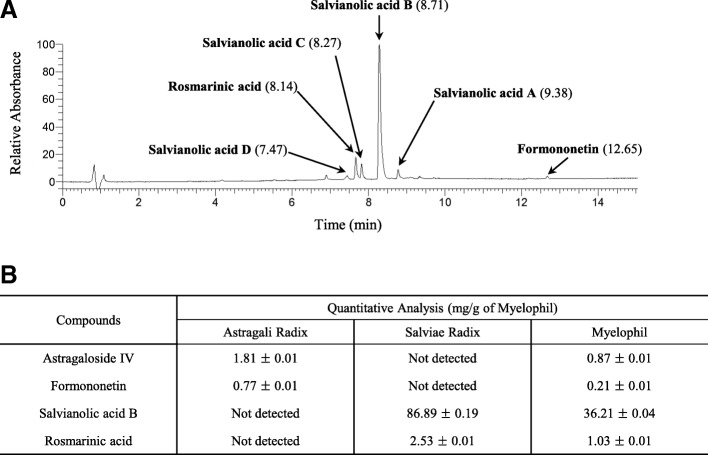
UHPLC and LC/MS chromatogram of Myelophil. Myelophil and reference compounds were subjected to UHPLC analysis (**a**). Myelophil and four major compounds (Astragaloside IV and Formononetin for *Astragali radix* and Salvianolic acid B and Rosmarinic acid for *Salviae Miltiorrhizae radix*) were quantified by LC/MS (**b**)

Myelophil was prepared in powder form by Kyung-Bang Pharmacy (Incheon, Korea) as follows according to the approved good manufacturing practice (GMP) guidelines of the KFDA [15]. Myelophil is the 1:1 mixture of *Astragali Radix* (Astragalus membranaceus Bunge, cultivated in Jecheon, South Korea Ser. No. 20101106-JC-HG) and *Salviae Miltiorrhizae Radix* (Salvia miltiorrhiza Bunge, cultivated in Hebei, China; Ser. NO. 20110302-CHN-DS). These two herbal materials were purchased from Daeyeon Pharmacy (Supplier of standardized herbs, Incheon, Korea), and they were confirmed by expert for herbology who is an herbal pharmacist. Myelophil was extracted using 30% ethanol for 20 h at 80 °C and the final product obtained with a yield of 20.52% (w/w) was stored for future use. To confirm the reproducibility of Myelophil’s components, ultra-high-performance liquid chromatography tandem mass spectrometry (UHPLC-MS/MS, Thermo Scientific, San Jose, CA, USA) method was re-conducted as described previously [16] (Fig. 1a). Liquid chromatography-mass spectrometry (LC/MS, LTQ ORbitrap XL linear ion-trap MS system, Thermo Scientific, San Jose, CA, USA) was also performed on the Myelophil as well as 4 reference compounds (astragaloside IV and formononetin for Astragali Radix and salvianolic acid B and rosmarinic acid for Salviae Miltiorrhizae Radix, respectively) for quantitative analysis as previously described [17] (Fig. 1b).

### Animals

A total of 40 beagle dogs (20 males and 20 females) were purchased from Woojung BSG (Gyeonggi-do Suwon, Korea) and used for the study. Each dog was acclimated to conditions in a stainless-steel mesh box (700 mm W × 750 mm L × 750 mm H) for 3 weeks and was subjected to routine examination daily. The environment was maintained at 21.9 ± 0.8 °C with a 12-h light/dark cycle, and the air was exchanged between 10 and 15 times/h. Each dog received 300 g/day of a standard dry diet (Biopia; Gyeoggi-do Gunpo, Korea) and had free access to automatically filtered tap water that had undergone a purification process. At the time of drug administration, the dogs averaged 6–7 months of age and ranged from 5.52 to 6.72 kg for males and 5.04 to 6.42 kg for females. All animals were checked for health before the drug administration. This study was conducted in Korea Conformity Laboratories (KCL, Incheon, Korea), an authorized institute for toxicological research, and adhered to the Testing Guidelines of KFDA [18]. Experts including histopathologist, animal specialist, and drug manager confirmed that this toxicity study was performed correctly.

### Acute toxicity

After an overnight fast, one male and one female in each of the four groups received 5,000 mg/kg, 2,500 mg/kg, 1,250 mg/kg, and 0 mg/kg doses of Myelophil using an oral capsule (single dose). Clinical signs were observed for 6 h after drug administration; thereafter, daily mortality and symptoms were observed for 2 weeks. Body weights were measured 1, 3, 7, and 14 days after administration, and necropsy was performed on day 14.

### Repeated toxicity

For the repeated toxicity test, 32 beagles (16 male and 16 female) were dived into 4 groups (Control: 10 dogs at 0 mg/kg, low dose: 6 dogs at 312.5 mg/kg, middle dose: 6 dogs at 625 mg/kg and high dose: 10 dogs at 1,250 mg/kg). Over 13 weeks, each dog was administered Myelophil by using an oral capsule once a day. In the two groups with 5 males and 5 females (1,250 mg/kg and 0 mg/kg), the extra 2 males and 2 females of each group were set up as recovery groups for 4 weeks.

Clinical signs of toxicity were checked once a day, and body weights and feed intake were measured once a week. Before the drug administration and within 1 week prior to the necropsy, ophthalmological examination, electrocardiography, urinalysis, hematological, and various biochemical parameters were performed. After necropsy, the external findings were recorded, and all organs, such as abdominal organs, thoracic organs and brain, were weighed. Histopathological examinations were performed for the following organs: brain, pituitary, heart, lungs, liver, gallbladder, kidney, bladder, mesenteric lymph nodes, thymus, spleen, pancreas, salivary glands, submandibular lymph nodes, thyroid, adrenal gland, esophagus, aorta, Spinal cord, sciatic nerve, skeletal muscles, skin, mammary gland, eye ball, stomach, pancreas, thymus, thyroid gland, parathyroid gland, duodenum, jejunum, ileum, appendix, colon, rectum, femur, sternum, trachea, tongue, prostate gland, testis, epididymis, ovary, bladder, uterus, and vagina.

### Statistical analysis

The analysis of the continuous data (organ weight, food intake, and hematological and biochemical parameters) was performed using one-way ANOVA. Statistical differences between the groups were analyzed using Dunnett’s multiple comparison test [19]. Dunnett’s t-test was performed when the dispersion was not homogeneous. The analysis of discontinuous data used the chi-square test after re-input of data after scale conversion. All analyses were performed using SPSS 12.0 K program, which is a widely used statistical package.

## Results

### Acute toxicity

No animals died during the test. In female animals, vomiting was observed on the day of administration in the 5,000 mg/kg group and the day after administration in the 2,500 mg/kg and 1,250 mg/kg groups. Compound-colored stools were observed on the 2nd day after administration in the 2,500 mg/kg group. These symptoms were also observed in the male animals; vomiting on the day of administration in the 5,000 mg/kg group and compound-colored stools in the 2,500 mg/kg group until day 4 after administration. There were no changes in body weight during the study period, and no abnormal lesions were observed at necropsy (Table 1).

**Table 1:** Summary of the acute toxicity test in beagle dogs

MYP (mg/kg)	0	1250	2500	5000
Number (M/F)	2 (1/1)	2(1/1)	2(1/1)	2 (1/1)
Mortality	–	–	–	–
Vomiting on the day of administration	–	–	–	++ (M, F)
Vomiting on the day after of administration	–	+ (F)	+ (F)	–
Compound-colored stool	–	–	+ (M)	–
Body weight change	–	–	–	–
Necropsy findings	NAD	NAD	NAD	NAD

Beagle dogs in each group were fed Myelophil (MYP). Clinical signs were observed for 6 h after drug administration; thereafter, daily mortality and symptoms were observed for 2 weeks. At the end of the experiment, necropsy was performed

*M* Male, *F* Female, *NAD* No abnormality detected; −: Absent; +: Slight; ++: Moderate

### Repeated toxicity

#### Clinical signs and mortality

No deaths were observed in any group during the study period. Drug compound-colored stools were observed in the 625 and 1,250 mg/kg groups, which appeared to be dose-related in both males and females. Soft stools were observed in all the dose groups (including the control) in a dose-related pattern in both males and females. Anorexia was sporadically observed all groups but without a dose-correlation (Table 2).

**Table 2:** Summary of the repeated toxicity test in beagle dogs

MYP (mg/kg per day)	0	312.5	625	1250
Number (M/F)	10 (5/5)	6(3/3)	6(3/3)	10 (5/5)
Mortality	–	–	–	–
Anorexia	−+	−+	−+	−+
Soft stool	+	+	++	+++
Compound-colored stool	–	–	++	+++
Food intake	NAD	NAD	NAD	NAD
Ophthalmological findings	NAD	NAD	NAD	NAD
Relative organ weight	NS	NS	NS	NS
Necropsy findings	NAD	NAD	NAD	NAD

Beagle dogs in each group were fed MYP, and the clinical symptoms, including mortality, were monitored for 13 weeks. At the end of the experiment, ophthalmological, necropsy-based examinations were performed

*NAD* No abnormality detected, *NS* Not significant; −: Absent; +: Slight; ++: Moderate; +++: Severe; −+: Sporadically detected

#### Weight change and feed intake

**Fig. 2:**
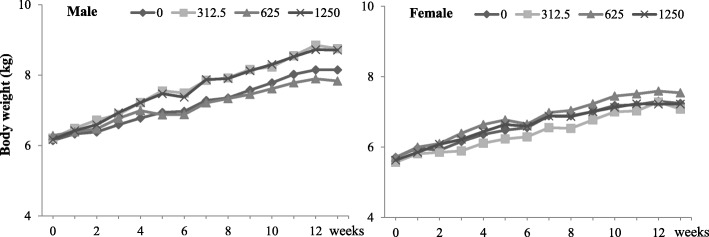
Body weight changes of dogs after 13 weeks. There were no significant differences in the body weight in the groups administered with Myelophil compared with the control group during the study period

Body weight increased gradually during the study period in all groups. However, there were no significant differences in the body weights or food intake in groups administered with Myelophil compared to the controls during the study period (Fig. 2).

#### Hematologic tests

At the 13th week of administration, no abnormal parameters were observed in the complete blood counts (CBC), including the blood-clotting time tests, in male or female animals of any group (Table 3).

**Table 3:** Hematological analysis after administration of Myelophil for 13 weeks

MYP (mg/kg)	Male				Female			
	0	312.5	625	1250	0	312.5	625	1250
Number	5	3	3	5	5	3	3	5
WBC (10^3^/μL)	9.4 ± 0.8	10.6 ± 2.2	10.0 ± 2.1	8.4 ± 1.3	7.6 ± 0.7	8.1 ± 1.6	8.7 ± 1.6	9.0 ± 1.7
WBC Differential Counts (%)								
NE	52.0 ± 3.5	54.3 ± 4.6	49.3 ± 8.7	62.1 ± 3.0	50.3 ± 8.1	55.5 ± 5.2	50.5 ± 4.0	50.1 ± 10.2
LY	36.8 ± 6.2	37.0 ± 4.2	41.5 ± 11.4	38.8 ± 2.4	39.2 ± 7.7	35.3 ± 6.7	41.9 ± 3.9	42.1 ± 10.5
MO	4.9 ± 1.2	4.0 ± 0.7	4.4 ± 1.9	4.5 ± 1.3	4.5 ± 0.8	5.0 ± 1.4	4.5 ± 0.9	4.0 ± 1.0
EO	5.8 ± 3.3	4.2 ± 1.1	4.3 ± 1.2	4.1 ± 0.9	5.3 ± 2.9	3.6 ± 1.2	2.3 ± 0.7	3.2 ± 1.3
BA	0.3 ± 0.2	0.4 ± 0.1	0.3 ± 0.2	0.4 ± 0.1	0.6 ± 0.2	0.4 ± 0.3	0.6 ± 0.2	0.5 ± 0.2
RBC (10^6^/μL)	7.3 ± 0.6	7.8 ± 0.3	7.5 ± 0.6	8.0 ± 0.3	8.3 ± 0.6	8.2 ± 0.8	8.4 ± 0.7	8.3 ± 0.7
Hemoglobin (g/dL)	16.3 ± 0.9	16.4 ± 0.6	16.0 ± 1.1	17.1 ± 0.9	18.5 ± 1.1	17.6 ± 1.2	18.9 ± 1.6	18.3 ± 1.2
Hematocrit (%)	49.6 ± 2.6	50.7 ± 1.4	49.7 ± 3.1	52.8 ± 2.4	57.4 ± 3.7	55.4 ± 3.8	57.6 ± 4.5	56.4 ± 3.9
MCV (fL)	68.2 ± 2.5	64.8 ± 2.1	66.7 ± 2.2	66.1 ± 3.2	69.0 ± 1.4	67.7 ± 1.8	68.4 ± 1.1	68.4 ± 1.7
MCH (pg)	22.5 ± 0.7	20.9 ± 0.8	21.4 ± 1.1	21.4 ± 1.3	22.3 ± 0.5	21.5 ± 0.7	22.4 ± 0.0	22.2 ± 0.7
MCHC (g/dL)	32.9 ± 0.2	32.3 ± 0.3	32.1 ± 0.5	32.4 ± 0.5	32.3 ± 0.4	31.7 ± 0.5	32.8 ± 0.5	32.4 ± 0.3
RDW (%)	14.1 ± 0.6	14.4 ± 0.2	14.7 ± 1.5	14.6 ± 0.9	13.4 ± 0.9	13.6 ± 0.4	13.8 ± 0.6	14.2 ± 1.1
Platelet (10^3^/μL)	327.0 ± 44.2	326.3 ± 68.3	380.7 ± 35.6	349.6 ± 73.9	301.8 ± 54.0	352.7 ± 87.8	329.0 ± 62.4	326.4 ± 52.9
MPV (fL)	16.3 ± 1.4	17.8 ± 1.1	17.0 ± 0.3	16.6 ± 1.3	16.5 ± 1.1	16.4 ± 2.4	17.2 ± 0.8	16.6 ± 0.7
Reticulocytes (%)	1.1 ± 0.3	1.1 ± 0.2	1.1 ± 0.6	1.0 ± 0.3	0.8 ± 0.2	1.0 ± 0.3	1.1 ± 0.3	0.9 ± 0.2
PT (sec)	6.0 ± 0.2	6.00 ± 0.1	6.0 ± 0.2	6.0 ± 0.2	6.0 ± 0.2	6.1 ± 0.1	6.3 ± 0.3	6.1 ± 0.2
aPTT (sec)	12.3 ± 0.7	12.1 ± 0.3	12.2 ± 1.0	12.2 ± 0.8	12.8 ± 1.3	12.9 ± 0.8	11.5 ± 1.5	12.5 ± 0.6

After administration of MYP for 13 weeks, hematology was analyzed to compare the MYP-treated groups to the control group

*WBC* White blood cell, *NE* Neutrophil, *LY* Lymphocyte, *MO* Monocyte, *EO* Eosinophil, *BA* Basophil, *RBC* Red blood cell, *MCV* Mean corpuscular volume, *MCH* Mean corpuscular hemoglobin, *MCHC* Mean corpuscular hemoglobin concentration, *RDW* Red cell distribution width, *MPV* Mean platelet volume, *PT* Prothrombin time, *APPT* Activated partial thromboplastin time

#### Biochemical tests

At the 13th week of administration, the serum concentrations of sodium and chloride were significantly decreased in the 312.5 and 1,250 mg/kg groups of females compared to the control group (*P* < 0.05). Those findings were not observed in the male groups. Other abnormalities in the biochemical parameters were not observed in the Myelophil-administered groups (Table 4).

**Table 4:** Serum chemistry analysis after administration of Myelophil for 13 weeks

MYP (mg/kg, Number)	Male				Female			
	0 (5)	312.5 (3)	625 (3)	1250 (5)	0 (5)	312.5 (3)	625 (3)	1250 (5)
AST (IU/L)	38.4 ± 9.6	48.0 ± 7.2	42.3 ± 12.1	47.8 ± 6.8	40.6 ± 5.1	44.0 ± 2.0	47.3 ± 16.4	44.0 ± 6.0
ALT (IU/L)	40.0 ± 10.2	37.7 ± 9.1	31.3 ± 6.0	47.8 ± 19.8	36.8 ± 5.7	37.0 ± 5.6	40.0 ± 14.8	40.4 ± 10.4
ALP (IU/L)	334.8 ± 97.7	494.0 ± 103.5	330.3 ± 116.6	345.2 ± 60.2	336.8 ± 120.0	219.3 ± 50.5	295.0 ± 10.4	289.4 ± 79.7
BUN (mg/dL)	16.6 ± 2.4	17.4 ± 2.6	15.0 ± 2.3	14.3 ± 1.6	17.4 ± 4.8	17.0 ± 1.7	14.4 ± 2.7	14.4 ± 2.4
Creatinine (mg/dL)	0.72 ± 0.03	0.66 ± 0.11	0.77 ± 0.06	0.82 ± 0.10	0.72 ± 0.13	0.63 ± 0.03	0.75 ± 0.05	0.72 ± 0.08
Glucose (mg/dL)	89.8 ± 7.3	79.3 ± 3.8	84.3 ± 7.8	84.4 ± 9.5	86.6 ± 4.0	84.7 ± 3.1	92.0 ± 3.0	86.4 ± 9.6
Total cholesterol (mg/dL)	184.0 ± 35.2	169.7 ± 27.2	151.7 ± 19.7	170.6 ± 37.3	160.0 ± 28.9	165.7 ± 34.6	150.3 ± 3.2	162.6 ± 32.2
Total protein (g/dL)	5.5 ± 0.4	5.6 ± 0.2	5.6 ± 0.4	5.9 ± 0.3	5.8 ± 0.3	5.7 ± 0.4	5.8 ± 0.2	5.8 ± 0.4
CPK (U/L)	242.6 ± 45.3	372.0 ± 152.2	307.0 ± 77.2	369.2 ± 122.9	256.0 ± 56.2	319.0 ± 79.7	362.3 ± 249.4	256.0 ± 75.6
Albumin (g/dL)	2.6 ± 0.2	2.7 ± 0.1	2.6 ± 0.1	2.8 ± 0.1	2.7 ± 0.3	2.7 ± 0.3	2.9 ± 0.2	2.9 ± 0.2
Total bilirubin (mg/dL)	0.04 ± 0.01	0.06 ± 0.02	0.04 ± 0.02	0.06 ± 0.03	0.07 ± 0.02	0.05 ± 0.02	0.06 ± 0.03	0.07 ± 0.02
A/G ratio	0.89 ± 0.19	0.94 ± 0.02	0.87 ± 0.14	0.88 ± 0.07	0.88 ± 0.07	0.93 ± 0.09	0.98 ± 0.05	0.99 ± 0.04
Triglyceride (mg/dL)	24.4 ± 3.6	44.0 ± 17.8	23.0 ± 4.0	30.4 ± 9.8	28.0 ± 2.9	28.0 ± 4.4	31.3 ± 6.1	30.2 ± 9.3
Calcium (mg/dL)	10.3 ± 0.4	10.4 ± 0.4	10.2 ± 0.5	10.6 ± 0.5	10.3 ± 0.4	10.2 ± 0.5	10.3 ± 0.4	10.2 ± 0.6
Inorganic phosphorus (mg/dL)	6.0 ± 0.7	6.0 ± 0.1	5.7 ± 0.7	5.8 ± 0.6	5.2 ± 0.4	4.9 ± 0.3	5.1 ± 1.3	4.8 ± 0.3
Chloride (mmol/L)	112.4 ± 1.5	112.3 ± 0.6	110.7 ± 1.5	111.0 ± 2.0	109.8 ± 10.3	107.0 ± 1.7*	109.7 ± 1.2	108.6 ± 0.9*
Sodium (mmol/L)	149.2 ± 1.3	148.0 ± 0.0	148.0 ± 1.0	148.0 ± 2.4	146.4 ± 1.5	144.0 ± 1.0*	148.0 ± 1.7	145.8 ± 0.8*
Potassium (mmol/L)	5.3 ± 0.3	5.4 ± 0.2	5.4 ± 0.2	5.7 ± 0.2	5.0 ± 0.4	5.3 ± 0.2	5.3 ± 0.2	5.1 ± 0.2

After the administration of MYP for 13 weeks, the serum chemistry was analyzed and compared to the control group

The data were expressed as the means ± sd

*AST* Aspartate aminotransferase, *ALT* Alanine aminotransferase, *ALP* Alkaline phosphatase, *BUN* Blood urea nitrogen, *CPK* Creatine phosphokinase, *A/G ratio* Albumin globulin ratio

*A statistical significance was considered if *P* < 0.05 compared with the vehicle control

#### Urinalysis

At the 13th week of administration, occult blood was increased in a statistically significant manner in 312.5 mg/kg males compared with the control group, and a statistically significant decrease was observed in the 312.5, 625, and 1,250 mg/kg females compared to the control group. These changes had no dose-relatedness or male-to-female correlations. No other abnormal parameters were observed in the urinalysis in the male or female animals of all groups (data not shown).

#### Gross autopsy findings

Adhesion between the left lobe and the medial lobe of liver was observed in 1 animal in the 625 mg/kg group, but there was no dose correlation or histopathological abnormality. No other abnormalities associated with the administration of Myelophil were observed. No changes associated with test drug administration were observed in organ weight. No abnormalities in either eye examination or electrocardiography were observed in any group before or after the test (data not shown).

#### Histopathologic examinations

**Fig. 3:**
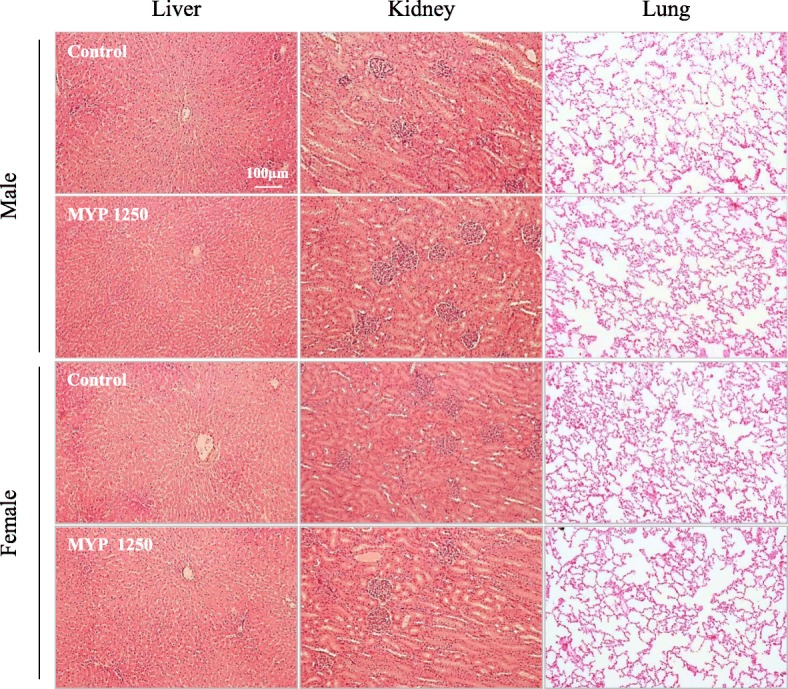
Histopathological findings. Representative pictures from H&E staining sections of the liver, lung, and kidney. No significant abnormalities associated with drug was observed

In all of the Myelophil-treated groups and control group, infiltrations of inflammatory cells in several organs (liver, lung, thyroid, testicle), white pulp atrophy of the spleen, vacuolization in interstitium of the kidney, degranulation in the thymus, and mineral deposition in the kidney were observed sporadically. All of these abnormalities were infrequent and were not related to the dose (Additional file 1: Table S1). The normal histopathological findings in the main organ (liver, lung and kidney) were shown in Fig. 3.

## Discussion

Our previous studies partially proved the safety of Myelophil in rats [11]. Rodent-derived studies are generally thought to provide only limited information on the adverse effects of drugs in humans [20]. In preclinical studies of drugs, the U.S. Food and Drug Administration (FDA) recommended that acute and repeated toxicity testing should be conducted in at least two mammalian species, including a non-rodent species [21]. In this study, we therefore evaluated the toxicity of Myelophil using beagle dog-based acute and repeated toxicological tests. Regarding the duration for repeated dose toxicity studies, the FDA generally recommends the same duration as is used for clinical purposes but a longer period than that required for the authorization of marketing [22]. For drugs used for more than 2 weeks and less than 1 month, FDA recommends a 3-month toxicity test in non-rodent. The common prescription-period of Myelophil is 4-week; therefore we designed a 13-week repeated toxicological test in our study.

Myelophil is a mixture of Astragali radix and Salvia radix extracts that has been prescribed to a wide spectrum of patients complaining of chronic fatigue and bone marrow dysfunctions in Daejeon University Hospital since 2002. This formula (the combination of *Astragali radix* and *Salviae Radix* extracts) was derived based on TCM theory to maintain the balance between *Qi* and *Blood*, and this combination is supported by experimental data that showed that it improved bone marrow function [8]. *Astragali radix* is ​​a medicinal herb that TCM indicates enhances *Qi* [23] and is reported to have immunomodulatory, anti-aging and antitumor effects [24–26]. *Salviae Radix* is a representative ​​herb that is used in TCM to treat blood-related disorders [27] and has been studied for antiplatelet aggregation, antioxidant, and anti-inflammatory effects [28–30].

In general, the clinical dose of Myelophil is 2,000–4,000 mg/day for a 60 kg adult. For evaluation of this clinical dose, we decided the maximum dose (1250 mg/kg) in repeated toxicological test based on NOAEL and safety factor (1/10) [31]. For the acute toxicity test, we determined the much larger maximum dose (5,000 mg/kg) to predict approximate lethal dose (ALD). In the present results, no beagle dog died following the single administration of 75 times the clinical dose of Myelophil (5,000 mg/kg), even in those dogs in which vomiting or drug compound-colored stool was observed. These symptoms were presumed to be due to the inability to absorb excess drug, and no abnormalities related to these side effects were found at the time of necropsy. Accordingly, the ALD was estimated to be greater than 2,500 mg/kg. The main purpose of a preclinical toxicological study is to evaluate the NOAEL value, which provides the safety range of a test drug in clinical practice [32]. The NOAEL values can be determined using a repeated toxicological test, and the resulting animal-derived value can be converted to a human equivalent dose (HED) [33]. These results provide key information on the range of clinical doses [34]. The calculation of the NOAEL is derived from the test material and metabolic rate in animals, and then the values can be converted into HED according to the comparison of body surface index and body weight between animals and human [35].

In our study, the 13-week repeated toxicity studies founded that the NOAEL of Myelophil in beagle dogs was greater than 1,250 mg/kg for both males and females. The soft compound-colored stools appeared to be a dose-related side effect in the testing groups; however, no weight changes or any histopathological findings were observed. These stool changes completely disappeared during the recovery period. Therefore, these effects were not considered to be toxicological changes but were presumed to be due to the excessive dose of testing materials. In the biochemical tests and urinalysis, electrolyte changes (sodium and chloride) and occult blood were observed in the test group. However, the range of changes was within the normal range and had no dose relatedness and no male-female correlation. These changes are likely to occur in normal beagle dogs and considered not to be related to Myelophil. Sporadic and rare histopathological findings, including the infiltration of inflammatory cells in the liver and lung, were observed in all groups including the control group, which would indicate accidental or spontaneous lesions independent of test substance administration. Abnormal stool formation and sporadic histopathological legions are generally common findings in repeated toxicological studies, even for edible materials [36–38]. The results described above may indicate that Myeloplil is very tolerable and safe for the tested animals, namely, beagle dogs.

In fact, several toxicological studies have been conducted on the individual herbs, *Astragali radix* or *Salviae Radix*. A subchronic toxicity study reported on the safety of Astragali radix extracted with an organic solvent in Sprague Dawley (SD) rats and beagle dogs up to 39.9 g/kg and 19.95 g/kg [39]. Another toxicological study (acute and subchronic) showed that the *Salvia radix* aqueous extract had a NOAEL of 5.76 g/kg in SD rats [14]. In these studies, the administration routes were intraperitoneal or intravenous injection, and their extraction conditions (organic solvent or water) were also different from our study (30% ethanol extract). Our toxicity study examined the combination of two common herbs and thus considered the possibility of drug-drug interactions in the toxicity, even though each drug is safe [40].

## Conclusion

In summary, the NOAEL of Myelophil was over 1250 mg/kg in beagle dogs, which corresponded to an HED of 694 mg/kg. This result provides evidence for the safety of Myelophil at a clinical dose, which is an oral administration of 2,000–4,000 mg/day for a 60 kg adult. The present study provided the ALD and NOAEL values of Myelophil and toxicological information for the combination of *Astragali radix* and *Salviae Radix*.

## Additional file

Additional file 1: **Table S1.** Summary of histopathological findings. (DOCX 18 kb)

## Data Availability

The datasets used and/or analyzed during the current study are available from the corresponding author on reasonable request.

## Abbreviations

ALD: Approximate lethal dose
CBC: Complete blood counts
FDA: U.S. Food and Drug Administration
HED: Human equivalent dose
KFDA: Korea Food and Drug Administration
MYP: Myelophil
NOAEL: No observed adverse effect level
SD rat: Sprague Dawley rat
TCM: Traditional Chinese medicine
UHPLC: Ultra-high-performance liquid chromatography
