# Supplementary material for: Acute and repeated toxicological study of Myelophil, an ethanol extract of a mixture of Astragali Radix and Salviae Miltiorrhizae Radix, in beagle dogs
Source: BMC Complement Altern Med. 2019 Jul 8;19:166. doi: 10.1186/s12906-019-2588-3 (PMC6615141; doi:10.1186/s12906-019-2588-3)
Supplement: Supplementary file 1 — Table S1. Summary of histopathological findings. (DOCX 18 kb) [file 12906_2019_2588_MOESM1_ESM.docx]

| **Table S1. Summary of histopathological findings** | | | | | | | | | |
| --- | --- | --- | --- | --- | --- | --- | --- | --- | --- |
| (mg/kg, N)  Organ | Male | | Female | | (mg/kg, N)  Organ | Male | | Female | |
|  | 0 (5) | 1250 (5) | 0 (5) | 1250 (5) |  | 0 (5) | 1250 (5) | 0 (5) | 1250 (5) |
| Eye ball | NSL | NSL | NSL | NSL | Kidney | NSL | NSL | NSL | 1, I.I(+2) |
| Cerebrum | NSL | NSL | NSL | NSL | Adrenal gland | NSL | NSL | NSL | NSL |
| Cerebellum | NSL | NSL | NSL | NSL | Heart, Aorta | NSL | NSL | NSL | NSL |
| Pituitary gland | NSL | 1, O.O | NSL | NSL | Thymus | 2, S.S.A(+1) | 2, S.S.A(+2),  D.E(+2) | NSL | NSL |
| Spinal cord,  Sciatic nerve | NSL | NSL | NSL | NSL | Thyroid | 2, S.S.A(+1) | NSL | NSL | NSL |
| Muscle | NSL | NSL | NSL | NSL | Parathyroid gland | NSL | NSL | 2, D.E(+1) | 1, I.I(+/-) |
| Trachea | 1, I.I(+/-) | NSL | NSL | NSL | Salivary gland | 2, I.I(+1), D | NSL | NSL | NSL |
| Lung | 3, I.I(+1) | NSL | 1, S.A(+1) | 1, I.I(+1) | Skin | NSL | NSL | NSL | NSL |
| Tongue | NSL | NSL | NSL | NSL | Mammary gland | NSL | NSL | NSL | NSL |
| Esophagus | NSL | NSL | 1, I.I(+/-) | NSL | Femur, Sternum | NSL | NSL | NSL | NSL |
| Stomach | 1, I.I(+/-) | NSL | NSL | NSL | Testis | 2, I.I(+1), C.T(+1) | NSL | - | - |
| Small intestine | NSL | NSL | NSL | NSL | Epididymis | NSL | NSL | - | - |
| Large intestine | NSL | NSL | NSL | NSL | Prostate | NSL | 1, I.I(+1) | NSL | NSL |
| Pancreas | NSL | NSL | NSL | NSL | Bladder | NSL | NSL | NSL | NSL |
| Spleen | NSL | A(+/-) | NSL | A(+/-) | Ovary, Uterus | - | - | NSL | NSL |
| Liver | NSL | NSL | NSL | NSL | Vagina | - | - | NSL | NSL |
| gall bladder | 2, I.I(+1) | 2, I.I(+1) | 2. I.I(+1) | 3, I.I(+1) | Lymph nodes | NSL | NSL | NSL | NSL |
| N: Number, NSL: No severe lesion finding, A: Atrophy, C.T: Cystic change, D.E: Degeneration, I.I: Inflammatory cell infiltration, S.S.A: Starry sky appearance, D: Diffuse, (+/-): very slight (+1): slight, (+2): mild, (+3): moderate, (+4): severed, O.O: Organ omission. | | | | | | | | | |
